# Lack of recovery in monocyte human leukocyte antigen-DR expression is independently associated with the development of sepsis after major trauma

**DOI:** 10.1186/cc9331

**Published:** 2010-11-19

**Authors:** Aurélie Cheron, Bernard Floccard, Bernard Allaouchiche, Caroline Guignant, Françoise Poitevin, Christophe Malcus, Jullien Crozon, Alexandre Faure, Christian Guillaume, Guillaume Marcotte, Alexandre Vulliez, Olivier Monneuse, Guillaume Monneret

**Affiliations:** 1 Hospices Civils de Lyon, Service de réanimation, Hôpital Edouard Herriot, 5 place d'Arsonval - 69437 Lyon Cedex 03, France; 2 Hospices Civils de Lyon, Laboratoire d'immunologie cellulaire, Hôpital Edouard Herriot, 5 place d'Arsonval - 69437 Lyon Cedex 03, France; 3 Hospices Civils de Lyon, Service de chirurgie d'urgence, Hôpital Edouard Herriot, 5 place d'Arsonval - 69437 Lyon Cedex 03, France

## Abstract

**Introduction:**

Major trauma is characterized by an overwhelming pro-inflammatory response and an accompanying anti-inflammatory response that lead to a state of immunosuppression, as observed after septic shock. Diminished monocyte Human Leukocyte Antigen DR (mHLA-DR) is a reliable marker of monocyte dysfunction and immunosuppression. The main objective of this study was to determine the relation between mHLA-DR expression in severe trauma patients and the development of sepsis.

**Methods:**

We conducted a prospective observational study over 23 months in a trauma intensive care unit at a university hospital. Patients with an Injury Severity Score (ISS) over 25 and age over 18 were included. mHLA-DR was assessed by flow cytometry protocol according to standardized protocol. Mann-Whitney U-test for continuous non-parametric variables, independent paired t test for continuous parametric variables and chi-square test for categorical data were used.

**Results:**

mHLA-DR was measured three times a week during the first 14 days. One hundred five consecutive severely injured patients were monitored (ISS 38 ± 17, SAPS II 37 ± 16). Thirty-seven patients (35%) developed sepsis over the 14 days post-trauma. At days 1-2, mHLA-DR was diminished in the whole patient population, with no difference with the development of sepsis. At days 3-4, a highly significant difference appeared between septic and non-septic patients. Non- septic patients showed an increase in mHLA-DR levels, whereas septic patients did not (13,723 ± 7,766 versus 9,271 ± 6,029 antibodies per cell, p = .004). Most importantly, multivariate logistic regression analysis, after adjustment for usual clinical confounders (adjusted OR 5.41, 95% CI 1.42-20.52), revealed that a slope of mHLA-DR expression between days1-2 and days 3-4 below 1.2 remained associated with the development of sepsis.

**Conclusions:**

Major trauma induced an immunosuppression, characterized by a decrease in mHLA-DR expression. Importantly, after multivariate regression logistic analysis, persistent decreased expression was assessed to be in relation with the development of sepsis. This is the first study in trauma patients showing a link between the lack of immune recovery and the development of sepsis on the basis of the standardized protocol. Monitoring immune function by mHLA-DR measurement could be useful to identify trauma patients at a high risk of infection.

## Introduction

The global burden of death and disability due to injuries is increasing, especially in patients younger than 40 years old [1]. In the course of supportive management, injured patients often develop sepsis, which is the most frequent cause of complications and death following severe injury [2]. Immunosuppression has emerged recently as a risk factor for sepsis in trauma patients [3,4]. It is now well established that any situation of injury or stress can induce a systemic inflammatory response that is often followed by an anti-inflammatory response [5-7]. This compensatory feedback mechanism, which maintains inflammatory immune homeostasis, is believed to lower natural defenses against pathogens and contribute to a state of immunosuppression [8-10] and is known to occur in cases of sepsis, septic shock, burns, stroke, and injury and in patients undergoing major surgery. Such alterations might be directly responsible for a detrimental outcome in trauma patients and for lowering the resistance to nosocomial infections in patients who have survived initial resuscitation [7-9,11].

In the absence of specific clinical signs of immune function in intensive care patients, biomarkers of immunosuppression are clearly highly desirable. Diminished expression of human leukocyte antigen DR expression on circulating monocytes (mHLA-DR) is widely accepted as a reliable indicator of immunosuppression in critically ill patients [12-14]. Some work has been devoted to trauma patients, but for the most part, these preliminary studies were performed 10 years ago (that is, before the advent of the last advanced trauma life support [ATLS] protocol for the management of multiple-injury patients). Early findings on mHLA-DR were based on limited numbers of patients and used non-standardized flow cytometry protocols [15-20]. The purpose of this study was to investigate mHLA-DR expression on the basis of the standardized protocol and to assess this expression as a predictive factor of infection in a multivariate analysis.

In the study described here, mHLA-DR expression was measured according to recently established flow cytometry protocols in a group of severely injured patients. The main objective of the study was to assess whether a low mHLA-DR expression might be a good predictor of infection in such patients.

## Materials and methods

### Patients' inclusion

This prospective observational study was carried out over a 15-month period (July 2008 to September 2009). The protocol was reviewed by the institutional ethics committee, which waived the need for informed consent because the study was observational and involved sampling of very small quantities of blood (100 μL). The purpose of the study was explained to the patients or members of their families. Samples were collected from residual blood after completion of routine follow-up.

Inclusion criteria were an Injury Severity Score (ISS) [21,22] of more than 25 and admission to the intensive care unit (ICU). Clinical exclusion criteria were age of less than 18 years, ISS of less than 25, chronic corticosteroid therapy, and death in the first 48 hours after admission. Patients admitted on a Saturday were excluded because mHLA-DR cannot be measured on day 1 or 2 (blood samples were not collected on Saturdays or Sundays, when the laboratory did not operate).

All patients admitted were followed up with prospectively until day 14 by daily clinical examination and biological tests. During follow-up, clinical and biological data were collected. The data collection comprised demographic characteristics (age and gender), infection characteristics (source, microorganisms identified, delay between trauma, and onset of sepsis), and outcome at 28 days (death or survival). Therapeutic data were also collected (a) on admission to the trauma room (the need for inotropic or vasoactive support and blood products [red blood cells, fresh frozen plasma, platelets, and albumin] and their quantities used to sustain a mean arterial pressure [MAP] up to 70 mm Hg [or 90 mm Hg in the case of cranial trauma], and the type and quantity of prophylactic antibiotics) and (b) during support (number of ventilator days, quantity and type of vasoactive support and of blood products, and use of massive transfusion, which was defined as more than 10 units of blood [23] or the replacement of the patient's total blood volume [24] over a 24-hour period). Creatinine, lactate concentration, and abnormal biphasic pulse transmittance waveform (BPW) were measured daily. Three clinical scores were recorded: ISS on admission (range of 0 to 75), initial severity of disease as assessed by the new Simplified Acute Physiology Score II (SAPS II) (range of 0 to 164) [25], and the Sepsis-related Organ Failure Assessment (SOFA) score (range of 0 to 24) on admission and every day during follow-up [26]. Severe brain and thoracic injury, which are well established as risk factors for sepsis development, were also taken into account [22].

### Sepsis definition

The American College of Chest Physicians/Society of Critical Care Medicine Consensus Conference [27] definition of sepsis was used for this study, namely the presence of an identifiable site of infection and evidence of a systemic inflammatory response on the basis of at least two of the following criteria: (a) body temperature of greater than 38°C or of less than 36°C, (b) heart rate of greater than 90 beats per minute, (c) respiratory rate of greater than 20 breaths per minute or hyperventilation as indicated by an arterial partial pressure of carbon dioxide (PaCO_2_) of less than 32 mm Hg (less than 4.3 kPa), and (d) a white blood cell count of greater than 12,000 cells/mm^3 ^or of less than 4,000 cells/mm^3 ^or the presence of more than 10% immature neutrophils. The onset of sepsis was defined, as recommended by the Consensus Conference [27], as the day on which the site of infection was identified. The final diagnosis of sepsis was retrospectively established by two experts assessing the complete medical data and not involved in case management. Diagnoses of pneumonia and urinary infection were established according to the guidelines of the American Thoracic Society and the Infectious Diseases Society of America [28] and of the Centers for Disease Control [29], respectively. Physicians were not informed of mHLA-DR results. BPW was also determined as it may be used as an indicator of sepsis development [30-32].

### Blood sampling and flow cytometric analysis

Ethylenediaminetetraacetic acid (EDTA)-anticoagulated blood samples were collected at 8 a.m. every 2 days after injury (on Mondays, Wednesdays, and Fridays) (that is, at days 1 and 2, days 3 and 4, days 5 and 6, days 7 and 8, days 9 and 10, and days 11 and 12). Flow cytometric (EPICS XL; Beckman Coulter, Inc., Hialeah, FL, USA) expression of monocyte HLA-DR was assessed on arterial, venous, or capillary blood. Blood samples were stored immediately at 4°C and stained within 2 hours after collection, in accordance with the standardization recommendations for mHLA-DR measurement [33,34]. Staining and cell acquisition were undertaken as described in the European standardized protocol. Monoclonal antibodies and their respective isotype controls were used according to the manufacturers' recommendations: fluorescein isothiocyanate (FITC)-labeled anti-CD14 (10 μL; Immunotech, Marseille France) and phycoerythrin (PE)-labeled anti-HLA-DR (20 μL; BD Pharmingen, San Diego, CA, USA) per 100 μL of whole blood. Monocytes were characterized on the basis of their CD14 expression. Results were expressed as the number of anti-HLA-DR antibodies per cell (AB/C) (normal >15,000), which is correlated with the number of HLA-DR molecules expressed on each monocyte [33].

Because sepsis alone can amplify a drop in mHLA-DR expression, mHLA-DR expression data were excluded from the analysis after the onset of sepsis, thereby precluding calculation of a difference in mHLA-DR expression between septic and non-septic patients at days 7 and 8, 9 and 10, and 11 and 12 (because of insufficient numbers of values for statistical analysis).

### Statistical analysis

The Kolmogorov-Smirnov test was used to verify all data for normality. Baseline characteristics were described by frequency, median and interquartile range (IQR), or (where appropriate) mean ± standard deviation. Patients were separated into two groups: those who developed sepsis and those who did not. The groups were compared using the Mann-Whitney *U *test for continuous non-parametric variables, the independent paired *t *test for continuous parametric variables, and the chi-square test for categorical data. mHLA-DR expression was stratified according to the best threshold chosen using the Youden index. Receiver operating characteristic (ROC) curves and the areas under the curve were calculated for the slope in mHLA-DR between days 1 and 2 and days 3 and 4. Univariate and multivariate logistic regression analyses were used to identify variables associated with the risk of infection and assessed by odds ratios (ORs) and 95% confidence intervals (CIs). A *P *value of less than 0.05 was taken as the significance level. The Bonferroni correction was used to avoid spurious results from the multiple statistical tests performed simultaneously. The alpha values for three or six tests were 0.016 and 0.008, respectively. MedCalc software version 9.6.4.0 (MedCalc Software bvba, Mariakerke, Belgium) was used to perform the statistical analyses.

## Results

### Patients' characteristics

**Figure 1:**
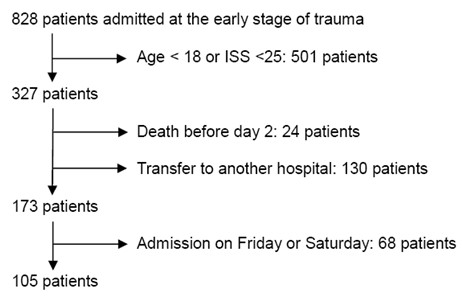
**Flowchart of inclusion criteria of the study**. ISS, Injury Severity Score.

A total of 536 consecutive patients in the early stages of trauma were admitted to the trauma room between July 2008 and May 2010. One hundred five of these patients met the inclusion criteria of the study (Figure 1). One hundred thirty patients were excluded because they had been rapidly transferred to another hospital for different reasons: no available rooms in our unit or the need for specific care such as aortic rupture isthmus or brain surgery (following severe brain injury). Table 1 shows the baseline characteristics on these 105 patients. SAPS II was significantly higher in septic patients (*P *< 0.05) than in non-septic patients as were the SOFA scores every day during follow-up and the incidence of severe brain injury. There were no statistical differences of the ISS or the incidence of severe thoracic injury between the two groups. In the emergency room, administration of vasoactive drug to maintain an MAP of up to 65 mm Hg and administration of prophylactic antibiotics were not different. Frequency of massive transfusion and the overall quantity of transfused blood were not different for the sepsis and non-sepsis groups over the first 2 post-trauma days. There was a higher proportion of patients under vasoactive drug during the first 2 days in the septic group (*P *= 0.0004). During follow-up, no difference in renal function (assessed by plasma creatinine concentration) or in lactate concentration was observed. Septic patients required mechanical ventilation more often and for longer periods of time than non-septic patients did (*P *< 0.0001). Six patients died (three from septic shock and three from cardiogenic shock), and there were no statistical differences between the two groups.

**Table 1:** Clinical patients' characteristics

Parameters	Overall population *n *= 105	Septic *n *= 37 (35%)	Non-septic *n *= 68 (65%)	*P *value
Age, years	38.1 ± 16.9	34.8 ± 15.6	39.9 ± 17.5	0.15^a^
Male, % (n)	72.4% (*n *= 76)	78.4% (*n *= 29)	69.1% (*n *= 47)	0.43^b^
ISS	37.1 ± 9.9	38.7 ± 8.9	36.2 ± 10.4	0.20^a^
Severe brain injury, % (n)	41% (*n *= 43)	59% (*n *= 22)	31% (*n *= 21)	0.008^b^
Severe thoracic injury, % (n)	72% (*n *= 76)	65% (*n *= 24)	76% (*n *= 52)	0.30^b^
SAPS II	36.9 ± 15.6	43 ± 15.4	33.5 ± 14.8	0.003^a^
Delay for MAP >65 mm Hg, minutes	0 (0 to 0.25)	0 (0 to 16.25)	0 (0 to 0)	0.14^c^
Need for vasoactive support in emergency room, % (n)	24% (*n *= 25)	35% (*n *= 13)	18% (*n *= 12)	0.077^b^
Prophylactic antibiotics administrated in emergency room, % (n)	42% (*n *= 44)	35% (*n *= 13)	46% (*n *= 31)	0.41^b^
SOFA score				
D1	4 (2 to 7)	6 (4 to 9.2)	3 (2 to 5)	0.0001^c^
D2	4 (2 to 6)	6 (3.75 to 9.25)	2.5 (1 to 5)	<0.0001^c^
D3	3 (1 to 5)	7 (3 to 9.25)	2 (1 to 3)	<0.0001^c^
D4	2 (1 to 5)	5 (2 to 8)	2 (1 to 3)	<0.0001^c^
D5	2 (1 to 4)	4 (1.75 to 7.25)	1 (1 to 2)	<0.0001^c^
D6	1 (1 to 3)	3 (1 to 7)	1 (1 to 2)	<0.0001^c^
mHLA-DR levels, antibodies per cell				
D1 and 2	11,371 ± 4,870	11,753 ± 4,291	11,177 ± 5,169	0.62^a^
D3 and 4	12,224 ± 7,501	9,271 ± 6,029	13,723 ± 7,766	0.004^a^
D5 and 6	15,623 ± 9,123	11,707 ± 6,004	16,602 ± 9535	0.05^a^
Variations in mHLA-DR, antibodies per cell				
D3 and 4/D1 and 2	1.25 ± 0.57	0.83 ± 0.43	1.44 ± 0.53	<0.0001^a^
D5 and 6/D3 and 4	1.37 ± 1.11	1.32 ± 0.82	1.38 ± 1.18	0.83^a^
Deaths at day 28, % (n)	6% (*n *= 6)	8% (*n *= 3)	4% (*n *= 3)	0.73^b^
Mechanical ventilation, % (n)	66% (*n *= 69)	89% (*n *= 33)	53% (*n *= 36)	0.0004^b^
Duration of mechanical ventilation, days	6 (3 to 11)	9 (6.75 to 19)	3 (2 to 5.5)	<0.0001^c^
Massive transfusion required, % (n)	29% (*n *= 31)	35% (*n *= 13)	26% (*n *= 18)	0.48^b^
Volume of transfusion, mL	900 (0 to 2,850)	1,200 (0 to 3,500)	0 (0 to 2,700)	0.067^c^
Shock (need for vasoactive drug on D1 and 2), % (n)	33% (*n *= 35)	57% (*n *= 21)	21% (*n *= 14)	0.0004^b^
Length of stay in ICU, days	9 (6 to 15)	15 (10 to 24.25)	7 (5 to 11)	<0.0001^c^

Parametric variables are expressed as mean ± standard deviation, and non-parametric variables are expressed as median (interquartile range) or frequencies. ^a^Independent samples *t *test; ^b^chi-square test; ^c^Mann-Whitney test. D, days; ICU, intensive care unit; ISS, Injury Severity Score; MAP, mean arterial pressure; mHLA-DR, monocyte human leukocyte antigen-DR; SAPS II, Simple Acute Physiology Score II; SOFA, Sepsis-related Organ Failure Assessment.

### Incidence of sepsis

Thirty-seven patients developed sepsis during follow-up. Pneumonia was the more frequent infection (*n *= 30), followed by urinary tract infection (*n *= 7). Causative bacteria were fairly evenly distributed between Gram-positive (*n *= 14) and Gram-negative (*n *= 21) organisms. Two patients had a mixed bacterial infection (Gram-positive and -negative). The median interval between trauma and onset of sepsis was 4 days (3 to 6.25).

### Monitoring of mHLA-DR expression

**Figure 2:**
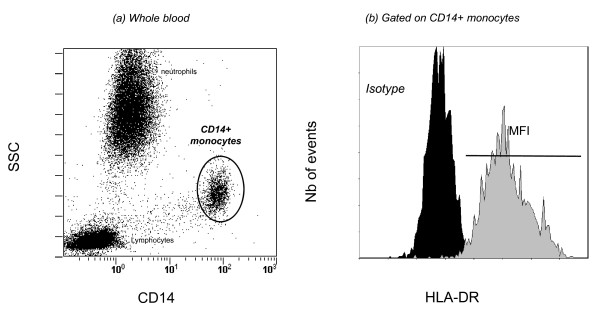
**Monocyte human leukocyte antigen-DR (HLA-DR) measurement by flow cytometry**. **(a) **Monocyte identification in whole blood. An ungated leukocyte biparametric representation on the basis of side scatter characteristics (SSC, y-axis) and CD14 expression (FITC-CD14, x-axis) is shown. CD14-expressing population is easily distinguishable as gating region 'CD14+ monocytes'. **(b) **Gated cells from 'CD14+ monocytes' in (a) are expressed on the basis of HLA-DR expression (monoparametric histogram, PE-HLA-DR). The black histogram depicts isotype control, whereas the gray one represents patient expression (illustrative example). Results are obtained as means of fluorescence intensities (MFI) and then are transformed into number of antibodies per cell (AB/C). FITC, fluorescein isothiocyanate; PE, phycoerythrin.

**Figure 3:**
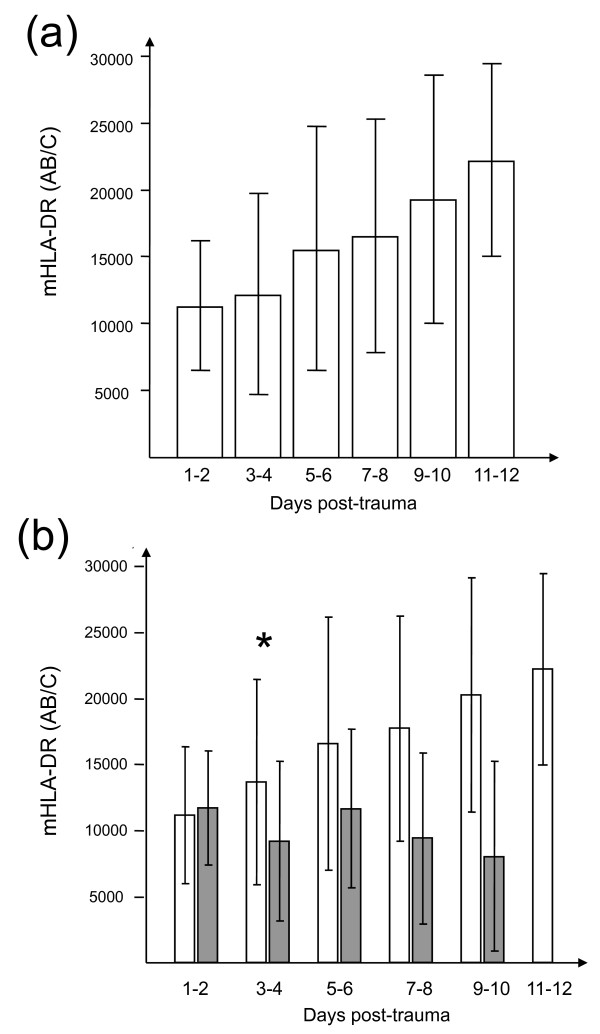
**Time course of monocyte human leukocyte antigen-DR (mHLA-DR) expression in trauma patients**. Mean and standard deviation are presented. Results are expressed as numbers of anti-mHLA-DR antibodies bound per cell (AB/C). The independent paired *t *test was used for comparison between groups. *P < 0.01. **(a) **mHLA-DR expression in the whole trauma population. **(b) **mHLA-DR expression in patients with (gray bars) or without (white bars) sepsis.

At day 2, mHLA-DR expression was diminished in all 105 patients (Figure 2 and 3a). At days 1 and 2, mHLA-DR expression showed no statistically significant difference between septic and non-septic patients (Table 1 and Figure 3b). At days 3 and 4, mHLA-DR expression had risen in non-septic patients but remained low in septic patients (13,723 ± 7,766 AB/C versus 9,271 ± 6,029 AB/C; *P *= 0.004) (Figure 3b). At days 5 and 6, septic patients still tended to exhibit lower mHLA-DR expression than non-septic patients, but the difference failed to reach statistical significance.

**Figure 4:**
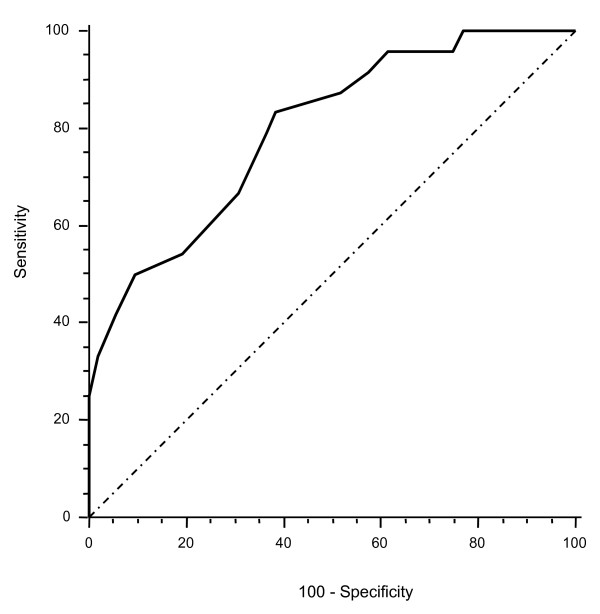
**Receiving operating curve of variation of monocyte human leukocyte antigen-DR expression ratio (days 3 and 4/days 1 and 2) expressed as antibodies per cell for predicting sepsis**. Area under curve was 0.80 (*P *= 0.05, 95% confidence interval 0.69 to 0.88). The best threshold (that is, which maximized sensitivity and sensibility) was 1.2. For a cutoff of 1.2, positive predictive value was 42% and negative predictive value was 87%.

Given the wide fluctuation of mHLA-DR expression data, ratios were calculated between values for two points in time, namely days 3 and 4/days 1 and 2 and days 5 and 6/days 3 and 4. The slope of mHLA-DR expression at days 3 and 4 showed a highly significant statistical difference between non-septic and septic patients (1.44 ± 0.53 versus 0.83 ± 0.43, respectively; *P *< 0.0001) (Table 1). We next established an ROC analysis (Figure 4). The area under the curve was 0.80 (*P *= 0.05, 95% CI 0.69 to 0.88). ROC curve analysis for delta mHLA-DR provided a 1.2 variation in mHLA-DR expression (days 3 and 4/days 1 and 2) as the best cutoff value to discriminate between septic and non-septic patients. At that threshold, the test had an 83% sensitivity, a 61% specificity, a 42% positive predictive value, and an 87% negative predictive value. At days 5 and 6, no significant difference was observed in mHLA-DR expression or in the mHLA-DR slope (days 5 and 6/days 3 and 4) between the two patient groups.

Multivariate logistic regression analysis indicated, after adjustment for other confounding factors, that an mHLA-DR ratio of days 3 and 4/days 1 and 2 less than or equal to 1.2 was associated with sepsis to a highly significant degree (adjusted OR 5.41, 95% CI 1.42 to 20.52) (Table 2).

**Table 2:** Univariate and multivariate logistic regression analysis used to differentiate septic and non-septic patients

		Univariate (*n *= 105)			Multivariate (*n *= 77)		
			
		OR	95% CI	*P *value	OR	95% CI	*P *value
Sex male	Positive	1.62	0.63-4.13	0.3129			
Severe brain injury	Positive	3.28	1.42-7.56	0.005	2.87	0.95-8.72	0.06
Severe thoracic injury	Positive	0.57	0.23-1.37	0.21			
ISS	≥40	2.19	0.95-5.06	0.066	2.84	0.88-9.16	0.08
SAPS II	≥37	3.17	1.38-7.32	0.007	2.01	0.67-6.08	0.21
D3 and 4/D1 and 2 mHLA-DR	≤1.2	4.81	1.45-16	0.009	5.41	1.42-20.52	0.013
Massive transfusion	Positive	1.5	0.63-3.57	0.35			

CI, confidence interval; D, days; ISS, Injury Severity Score; mHLA-DR, monocyte human leukocyte antigen-DR; OR, odds ratio; SAPS II, Simple Acute Physiology Score II.

## Discussion

Severe injury is characterized by a systemic inflammatory response syndrome involving activation of several cellular systems and the overwhelming production, by the innate immune system, of proinflammatory cytokines and other inflammatory mediators. It is subsequently followed by a compensatory anti-inflammatory response syndrome [35,36]. This negative feedback mechanism has a protective effect over the first few hours after trauma but may become harmful if it persists. Considerable clinical and experimental evidence indicates that in such patients a number of immune functions are rapidly altered. Monocyte alterations, for example, can decrease phagocytosis, inflammatory cytokine expression, and antigen presentation because of the loss of mHLA-DR expression. Lymphocyte anergy and apoptosis can ensue [3,37-39]. These changes together may increase susceptibility to infection, which in turn could provoke multiple organ failure and death.

Diminished mHLA-DR expression has been proposed as a reliable biomarker of immunosuppression in ICU patients. Today, it is the most reliable marker and is used in most of the studies about ICU-acquired immunosuppression. More specifically, it has been shown to be a predictor of septic complications in several conditions, including surgical interventions, sepsis, burns, stroke, and pancreatitis [11,40-48]. Immunosuppression has long been postulated as a concomitant of trauma [37,49,50]. In regard to mHLA-DR, the pioneering work of Polk and colleagues [50] reported in 1986 revealed an association between the development of sepsis and low mHLA-DR expression. Subsequently, mHLA-DR expression was assessed as a predictor of sepsis in several series of severely injured patients [15,17,18,20,39,51-53]. A major limitation of these studies is that they were conducted over a 20-year period, during which time case management and methodologies for measurement of mHLA-DR expression have evolved, thereby complicating interpretation and comparison of the findings of these studies.

In the present cohort, incidence of sepsis was 35% and the mortality rate for the entire study was 6%. Though apparently high, these values are in concordance with those of a previous epidemiologic study by Osborn and colleagues [2], in which incidence rates of sepsis were 42% for moderate injury (defined as an ISS of between 15 and 29) and 39% for severe injury (ISS of up to 30). Another epidemiologic study in trauma patients reported a low sepsis incidence, but most of the patients presented with mild injury (ISS of less than 15: 67.7%) and no brain injury [54], the latter of which is known to be a risk factor for developing pneumonia [55-58]. In our cohort, septic patients presented more trauma brain injury than the non-septic patients did, and this is in concordance with the literature.

The present study showed an overall reduction in mHLA-DR expression in trauma patients. Most importantly, in injured patients with an uneventful outcome, mHLA-DR expression returned to normal within a week. In contrast, in patients who developed infection, mHLA-DR levels remained low or fell even lower.

It would appear that the steepness of the slope of mHLA-DR recovery is a more significant indicator than the levels attained at a given point in time. Indeed, the incidence of sepsis was significantly greater in the group with a slope of less than 1.2 (days 3 and 4/days 1 and 2). This suggests that patients in whom recovery in mHLA-DR expression did not begin between days 1 and 2 and days 3 and 4 had an increased risk of developing sepsis. This observation is consistent with recent findings reported by Lukaszewicz and colleagues [59] in surgical patients. Significantly, in the present study, multivariate logistic regression analysis indicated that low mHLA-DR expression was independently associated with the development of sepsis, whereas all the other parameters included in the analysis (ISS, SAPS II, presence of a severe brain trauma, and massive transfusion) were not predictive. A slope of mHLA-DR of less than 1.2 was independently associated with the risk of developing sepsis, a finding that reflects the possible pivotal role of immune dysfunction in the increased risk of infection in trauma patients. In Table 1 some variables that may seem relevant (like the length of stay in the ICU and the duration of mechanical ventilation) were not included in the multivariate analysis, because they have to be considered as a consequence of the development of sepsis and not a risk factor. Moreover, it has to be considered that the onset of sepsis is early (median at day 4) and that every patient was still in the ICU at this time point.

Chest trauma is reported to account for one third of acute-trauma emergency room admissions, and 30% to 75% of trauma patients have pulmonary contusions [60], usually as a result of rapid deceleration [61]. The incidence of lung injury seemed to be quite significant in our study (72%) and is probably due to the severity of the trauma patients included. However, there were no differences between the presences of thoracic injury between infected and non-infected groups. The pathophysiology of pulmonary contusion includes a strong inflammatory response in the lung parenchyma, resulting in increased alveolocapillary permeability, pulmonary edema, ventilation/perfusion mismatch, increased pulmonary shunting, and loss of compliance. As at the systemic level, this local response is followed by an anti-inflammatory response. Muehlstedt and colleagues [19] observed not only altered HLA-DR expression on the surface of alveolar macrophages in the lungs of trauma patients who developed sepsis but also altered production of other cytokines. Local organ immunosuppression was present and may have been responsible for the development of nosocomial pneumonia in the injured patients [19].

As far as the authors can ascertain, this is the first study, using the standardized test described by the European multicenter study [33] and multivariate analysis, aimed specifically at evaluating mHLA-DR expression in a cohort of severe trauma patients. Most previous studies have been conducted in smaller series of patients spanning a highly variable spectrum of severity (from mild to severe trauma) [15-17,58]. Furthermore, these studies did not include multivariate analysis for assessment of the usual clinical confounders of mHLA-DR expression levels, nor did they exclude mHLA-DR expression data following onset of sepsis, as the present study did (see Materials and methods) in order to avoid bias from possible amplification by the sepsis itself of lowered mHLA-DR expression. Finally, the data from previous studies are not readily comparable, owing to differences, from one study to another, in the values studied (mainly 'percentages of positive monocytes' or 'mean fluorescence intensity'), which are generally specific for a given laboratory and therefore defy comparability on a wider scale. The European protocol now recommends expression of the results as numbers of antibodies per cell, a recommendation that will facilitate comparison of data obtained by different laboratories.

The present study has a number of limitations. First, it is a single-center study. The findings clearly need to be confirmed by a multicenter study. Second, the study enrolled only 105 patients. Though relatively small, the series was very homogeneous in terms of severity and also highly representative of the trauma patient population commonly encountered. Finally, mHLA-DR expression was measured every 2 days after trauma. However, the mean onset of infection was on day 4; in some patients, this limited the amount of analyzable mHLA-DR expression data available before day 4. In subsequent studies, follow-up of patients should consist of daily monitoring during the early post-trauma period. Indeed, one potentially interesting objective of a future study would be an assessment of the usefulness of daily mHLA-DR measurements to detect patients at an increased risk of infection. To pre-empt development of infection, clinicians could give these patients prophylactic treatment, such as antibiotics [48], immunostimulant by interferon-gamma [62], or granulocyte-macrophage colony-stimulating factor, as used in septic shock [35].

## Conclusions

Trauma induces a temporary, relative immunosuppression characterized by diminished mHLA-DR expression. The pattern of progression of mHLA-DR expression over time appears to be a more useful indicator of increased risk of infection than the actual levels of mHLA-DR expression at given points in time. Patients in whom recovery of mHLA-DR expression begins after days 1 and 2 are likely to have an uneventful outcome, whereas those with persistently lower levels of mHLA-DR expression are more likely to suffer infection. Large, multicenter studies are needed to confirm these promising results.

## Key messages

• Severe trauma patients present with a transient immunosuppression with decreased mHLA-DR expression.

• The lack of mHLA-DR recovery between days 3 and 4 and days 1 and 2 is associated with sepsis.

• After adjustment for classic confounding risk factors, the lack of mHLA-DR recovery was the sole factor independently and significantly associated with the development of sepsis.

## Abbreviations

AB/C: antibodies per cell; BPW: biphasic pulse transmittance waveform; CI: confidence interval; ICU: intensive care unit; ISS: Injury Severity Score; MAP: mean arterial pressure; MHLA-DR: monocyte human leukocyte antigen-DR; OR: odds ratio; ROC: receiver operating characteristic; SAPS II: Simplified Acute Physiology Score II; SOFA: Sepsis-related Organ Failure Assessment.

## Competing interests

The authors declare that they have no competing interests.

## Authors' contributions

AC helped to design the study, collected the clinical information, analyzed the raw data, performed statistical analysis, drafted the paper, and contributed to the writing of the paper. BF helped to design the study and to include patients, participated with AC in the interpretation of all data, and contributed to the writing of the paper. BA and GMo helped to design the study, participated with AC in the interpretation of all data, and contributed to the writing of the paper. CaG, FP, and CM helped to perform the experiments. JC, AF, ChG, GMa, AV, and OM helped to include patients. All authors read and approved the final manuscript.
